# 5.O. Workshop: Psychosocial impact and healthcare utilization after terrorist attacks

**DOI:** 10.1093/eurpub/ckac129.334

**Published:** 2022-10-25

**Authors:** Organised by: EUPHA-PMH, Chair persons: Johan Bilsen (EUPHA-PMH), Jutta Lindert (EUPHA-PMH)

## Abstract

Terrorist attacks can be regarded as a major public health issue, confronting not only political leaders with adequate answers to these threats, but also the public healthcare, and especially the public mental healthcare. The impact of terrorist attacks is mostly severe, widespread, affecting those directly exposed and their close ones, but also first responders, healthcare workers, local communities and even the society at large. While the directly involved are often confronted with severe physical and acute, often transient, traumatic stress symptoms, longitudinal studies increasingly show that a much larger group is affected by persistent mental health problems, such as posttraumatic stress disorders, depression, or a general undermining of their mental and physical wellbeing, sometimes seriously impairing their personal and social functioning. These problems may differ by severity of exposure and individual risk factors, but are also related to access to timely, appropriate and effective healthcare and psychosocial support. The mostly unpredictability of these threatening events, the urgency of response, and the often-chaotic circumstances are challenging factors to identify the most vulnerable people that need psychosocial care, and to organize optimal care. It also impedes the assessment of the efficiency of implemented psychosocial care. However, systematic planning and evaluating psychosocial care based on scientific evidence on the best practices is important to efficiently respond to and recover from mass casualty incidents such as terrorist attacks. Until now, scientific knowledge about the current state of utilization of healthcare by the affected population, and about the best practices taking into account specificity of certain vulnerable subgroups is still very scarce. In this workshop four speakers from different countries will present their research findings about the psychosocial impact of terrorist attacks, the way how this is addressed by society and the actual healthcare utilization of the affected people. Recommendations regarding a more adequate response are proposed. Roel Van Overmeire will talk about the long-term psychosocial care response by the Belgium government, from both the level of the policy, as well as from the perspective of victims. Lise Stene will present a register-based study of survivors'utilization of primary care and mental health services before and after the Utoya attack in Norway. Yvon Motreff will inform us about the psychological impact on first responders and their engagement in mental health care after the 2015 terrorist attacks in France. Finally, Ulrich Wesemann will further focus on mental healthcare provided to emergency responders after the Berlin terrorist attack in 2016 in Germany, and will specifically examine the outcome of crisis intervention in relation to gender and occupational characteristics.

**Key messages:**

• Public mental health impact of terrorist attacks is substantial and needs an adequate and differentiated psychosocial care response.

• Research on healthcare utilization after terrorist attacks is needed to strengthen public health preparedness.

